# Explorando la Empatia de Estudiantes de primer semestre de Enfermería mediante el Empathy Quotient (EQ)*

**DOI:** 10.15649/cuidarte.1954

**Published:** 2022-10-14

**Authors:** Javier Hernando Jerez-Jaimes, Zuly Andrea Rodriguez-Corredor, Eliana Ximena Narvaez-Parra

**Affiliations:** 1 Universidad Cooperativa de Colombia, Bucaramanga, Colombia. Email: javjerez@gmail.com Universidad Cooperativa de Colombia Universidad Cooperativa de Colombia Bucaramanga Colombia javjerez@gmail.com; 2 Universidad Cooperativa de Colombia, Bucaramanga. Colombia. Email: zuly.rodriguezc@campusucc. edu.co Universidad Cooperativa de Colombia Universidad Cooperativa de Colombia Bucaramanga Colombia zuly.rodriguezc@campusucc.edu.co; 3 Universidad de Santander UDES, Bucaramanga, Colombia. Email: enarvaez@udes.edu.co Universidad de Santander Universidad de Santander Bucaramanga Colombia enarvaez@udes.edu.co

**Keywords:** Adolescente, Cuidados de Enfermería, Clase social, Empatía, Sexo, Adolescent, Nursing Care, Social Class, Empathy, Sex, Adolescente, Cuidados de Enfermagem, Classe social, Empatia, Sexo

## Abstract

**Introducción:**

La empatía ya sea considerada como una dimensión o una competencia, es crucial para el desarrollo de nuestra especie social, especialmente en una situación tan particular como el cuidado de otros.

**Objetivo:**

Determinar el cociente de empatía de los estudiantes de primer semestre de Enfermería de la Universidad Cooperativa de Colombia sede Bucaramanga.

**Materiales y Métodos:**

Se analizó el cociente de empatía mediante el test EQ de Baron-Cohen y Wheelwright en la totalidad de estudiantes del primer semestre (N: 100). Se determinó la relación con las variables sexo, edad, estrato social y procedencia, mediante pruebas de t y de F, así como ANOVA y Kruskal-Wallis.

**Resultados:**

No se encontraron diferencias estadísticas entre los cocientes de empatía de los sexos, edad, estrato social o procedencia, pero sí variaciones en la distribución de los datos. Se determinó una relación negativa entre la edad y el estrato social con los cocientes de empatía.

**Conclusiones:**

La media del cociente de empatía de la población de estudiantes de enfermería de primer semestre fue de 40,3 ubicándola en una posición media, el grupo de estudio fue bastante heterogéneo, con un desarrollo de empatía bajo a medio. Se hace necesario generar estrategias a lo largo de la carrera profesional para el desarrollo de la empatía en esta población de estudiantes, ya que la gran mayoría obtendrá su título profesional antes de alcanzar la madurez cerebral.

## Abstract (en)

**Introduction:**

Empathy either considered as a dimension or a competence is crucial for the development of our social species, particularly in a situation as special as the care of others.

**Objectives:**

To determine the quotient of empathy of the first semester students of nursing at the Universidad Cooperativa de Colombia hosts Bucaramanga and its relations with the variables sex, age, social condition and city of origin.

**Materials and Methods:**

We analyzed the quotient of empathy using the EQ test Baron-Cohen and Wheelwright in all students in the first semester (N: 100). The relationship with the variables sex, age, social stratum and origin were determined by t test and F test, as well as ANOVA and Kruskal-Wallis test.

**Results:**

No statistical differences were found between the quotients of empathy of the sexes, age, social condition or city of origin, but variations in the distribution of the data were observed. Was determined a negative relationship between age and social condition with the quotients of empathy.

**Conclusions:**

The mean of the quotient of empathy of the population of nursing students from first semester was of 40,3 locating it in a middle position, the study group was quite heterogeneous, with a development low to medium of empathy. Strategies are necessary throughout his professional career for the development of empathy in this population of students since the vast majority will get your professional degree before reaching the brain maturity.

## Resumo

**Introdujo:**

A empatia, seja considerada uma dimensáo ou uma competencia, é crucial para o desenvolvimento da nossa espécie social, sobretudo numa situado táo particular como o cuidado com os outros.

**Objetivo:**

Determinar o quociente de empatia de estudantes de enfermagem do primeiro semestre da Universidad Cooperativa de Colombia, Bucaramanga.

**Materiais e Métodos:**

O quociente de empatia foi analisado pelo teste Baron-Cohen e Wheelwright EQ em todos os alunos do primeiro semestre (N: 100). A relacáo com as variáveis sexo, idade, condicáo social e procedencia foi determinada por meio dos testes t e F, ANOVA e Kruskal-Wallis, além de Análise Canónica (AC).

**Resultados:**

Náo foram encontradas diferencas estatísticas entre os quocientes de empatia dos sexos, idade, estrato social ou procedencia, mas houve variares na distribuido dos dados. Uma relacáo negativa foi determinada entre idade e status social com quocientes de empatia.

**Conclusoes:**

A média do quociente de empatia da populado de estudantes de enfermagem no primeiro semestre foi de 40,3, o que a coloca em posido intermediária, sendo o grupo de estudo bastante heterogéneo, com baixo a médio desenvolvimento da empatia. É necessário gerar estratégias ao longo da carreira profissional para o desenvolvimento da empatia nessa populado de alunos, pois a grande maioria obterá seu diploma profissional antes de atingir a maturidade cerebral.

## Introducción

La “recompensa” es una tendencia típica en las especies sociales que permite asegurar con esto el alimento, el sexo, el cuidado y la socialización. La recompensa referida anteriormente no se define en este artículo en un contexto material, si no en sentido relacionado con el bienestar obtenido al hacer el bien. Para comprender la importancia y el origen de la empatía, se debe considerar que la especie humana (Homo sapiens) había sido considerada en stricto sensu agresiva por algunos etólogos como Konrad Lorenz, basado en las guerras mundiales y demás conflictos bélicos en la historia de la humanidad, en este sentido, animales incapaces de socializar o de empatizar. Hoy en día se considera a los humanos como seres capaces de cooperar y con comportamientos prosociales^1^, estando estos últimos relacionados con la conducta voluntaria y beneficiosa hacia los demás, involucrados con el desarrollo emocional y la personalidad, además de comprender acciones de ayuda, cooperación y altruismo^2^. Los mamíferos exhiben ejemplos de empatía por sus compañeros, un chimpancé pondrá su mano en el hombro de otro chimpancé que haya perdido una pelea. Los perros manifiestan su tristeza acercándose y recostándose junto a su padre o madre adoptivo cuando reconocen que éstos están enfermos o deprimidos, de estas amplias expresiones de empatía, surge la utilidad de los animales en programas de intervención clínica con ellos (Jerez-Jaimes, J y Narváez-Parra, E. Observación Personal. 2019). Los Bonobos (Pan panyscus) expresan tendencias sociosexuales de consolación por medio de contactos genitales, esta especie presenta mayor cantidad de materia gris en las regiones cerebrales involucradas en la percepción de angustia (amígdala dorsal derecha, ínsula anterior derecha) y un mejor circuito inhibidor de la agresión^3^.

**Figura 1:**
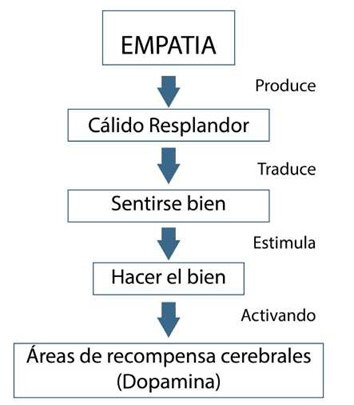
Efecto en cascada de la empatía

La empatía en los humanos es descrita como “tomar la perspectiva de otro o imaginarse a uno mismo en la posición del otro”. Otra forma de ver la empatía es como un fenómeno multicapa que comienza con un “estado automático de ajuste” basado en la imitación motora y representaciones neurales compartidas^4^. Se presupone un origen de la empatía a partir del cuidado maternal en mamíferos y esto explicaría los niveles y efectos de la oxitocina en los dos sexos^5^. La empatía produce un efecto de recompensa en quien la experimenta (Figura 1), generando una sensación de bienestar y el deseo de hacer el bien. De igual forma, la empatía cubre las vías en las cuales el estado emocional de uno afecta a los otros^1^.

Fuente: Elaborado por Javier Jerez-Jaimes, basado en Harbaugh et al^1^

### Niveles de la Empatía

**Figura 2:**
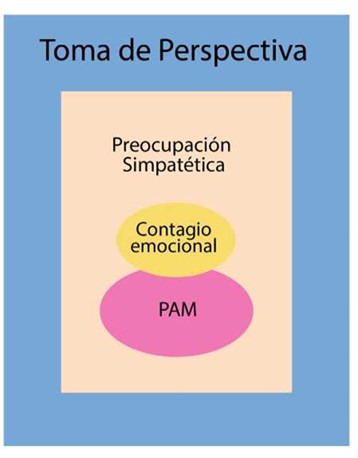
Modelo de capas de la empatía

Se han definido cuatro niveles en el proceso de empatía (Figura 2):
*El contagio emocional:* se refiere a la conexión emocional en la que una parte es afectada por el estado emocional del otro. En una jauría si un perro ladra, los otros lo seguirán. En una sala de recién nacidos humanos, si un bebé llora, automáticamente los demás empezarán a llorar.
*Preocupación simpatética:* la simpatía se define como una respuesta afectiva que consiste de sentimientos de tristeza o preocupación por otro miembro angustiado (diferente a compartir la emoción del otro). También se considera una motivación altruista. El mejor ejemplo de preocupación simpatética es la Consolación.
*Toma de perspectiva empática:* la capacidad para tomar la perspectiva del otro, la situación para tomar la perspectiva del otro, entendiendo la situación y necesidad específica del otro, separándolas de las propias.
*Toma de perspectiva empática:* la capacidad para tomar la perspectiva del otro, la situación para tomar la perspectiva del otro, entendiendo la situación y necesidad específica del otro, separándolas de las propias.
*Mecanismo de Acción Percepción PAM*^4^*:* en el núcleo de la capacidad empática hay un mecanismo que suministra a un observador (Sujeto) acceso al estado subjetivo del otro (Objeto) mediante las representaciones corporales y neurales del sujeto. Cuando el sujeto asiste al estado del objeto las representaciones neurales del sujeto de estados similares son automática e inconscientemente activadas.

*Elaborado por Javier Jerez-Jaimes, basado en de Waal*^6^

### Empatía en Enfermería

Según Magpantay-Monroe^7^ el estímulo de la inteligencia emocional (IE) en las enfermeras es importante para que ellas desarrollen determinación, integridad y compasión. La IE comprende tres dominios: el primero es la conciencia de sí mismo, el segundo es la autogestión y el tercero es la práctica de la empatía. La empatía se considera una de las características más importantes de las enfermeras ya que permite la comprensión de los pensamientos y sentimientos de los pacientes a través de la observación, la memoria, el conocimiento y el razonamiento^8^.

Evaluar la empatía en los profesionales de enfermería no es un tema nuevo, de hecho, la empatía y la compasión que la(o)s enfermera(o)s muestran a sus pacientes produce efectos benéficos en su recuperación. La frase “Efecto Nightingale” o “Síndrome Nigthingale” tiene que ver con las emociones y sentimientos que enfermera(o)s y pacientes pueden experimentar entre ellos. Se desconoce su origen, pero algunos sitios de internet hacen mención a la película Volver al futuro de 1984 pronunciada por el Dr. Browm a Marty y su mamá.

La empatía es definida como la capacidad para a) sentirse afectado y compartir el estado emocional de otro, b) evaluar las razones del porqué del estado del otro, c) identificarse con el otro asumiendo su perspectiva^6^. Loomis y de Natale^9^ concluyen que es posible que las enfermeras aprendan a tener empatía por pacientes en situaciones de vulnerabilidad y defender los derechos de estas personas. Ouzouni y Nakakis^10^ realizaron un estudio exploratorio de la empatía en estudiantes de enfermería en Grecia utilizando como instrumento evaluador el Jefferson Scale of Nursing Students Empathy (JSE) encontrando un moderado nivel de empatía, a su vez reportan que las hembras son ligeramente más empáticas que los machos y que los estudiantes de sexto semestre muestran un mejor nivel de empatía que los de primer semestre. Díaz-Narváez et al.,^11^ evaluaron la empatía en estudiantes de enfermería de la Universidad Mayor de Chile aplicando el JSE, encontrando que no había diferencia entre sexos y que la empatía se construía a medida que avanzaban en el desarrollo de la carrera.

En España Giménez-Espert et al.^12^ evaluaron los niveles de empatía de enfermeras profesionales utilizando como instrumento el JSE, reportando niveles altos de empatía en estas profesionales españolas. Triana-Restrepo^13^ midió los niveles de empatía en el Hospital Santa Clara en Colombia, principalmente de la relación enfermera-paciente, contra los niveles de ansiedad del paciente y encontró que, si se capacita a las enfermeras en la teoría de Peplau, los niveles de stress de los pacientes disminuyen y el nivel de empatía de las enfermeras aumenta.Es importante conocer el nivel de empatía de los estudiantes de primer semestre de los programas del área de salud humana o animal para evaluar su desarrollo a través de su formación y construir estrategias de refuerzo de esta competencia o dimensión. Por esta razón nos propusimos evaluar los niveles de empatía de los estudiantes de primer semestre de la Facultad de Enfermería de la Universidad Cooperativa de Colombia sede Bucaramanga y correlacionarlos con las variables edad, sexo, estrato social o ciudad de procedencia, para resolver los interrogantes básicos ¿Cuál es el rango de empatía de la población de ingreso al programa de Enfermería? ¿Son las mujeres más empáticas? ¿Influye la edad en el nivel de empatía? Y finalmente saber si ¿el lugar de procedencia Área metropolitana u otras ciudades influye en los niveles de empatía globales?

## Materiales y Métodos

La matriz de datos colectados y analizados se encuentra en Mendeley Data de acceso público, el archivo se encuentra en Excel Office identificado como: “Empatía y variables asociadas estudiantes primer semestre enfermería”^14^.

### Diseño de la experiencia pedagógica o proyecto de aula

Esta experiencia se diseñó pensando en el análisis de los datos obtenidos, por lo que, se puede definir como de tipo cuantitativa, descriptiva y transversal. Es claro, que no hubo modificaciones en las variables ni en el entorno de muestreo.

### Población

En este ejercicio pedagógico participaron la totalidad de estudiantes (N= 100) matriculados en el primer semestre del año 2020 en el programa de Enfermería de la Universidad Cooperativa de Colombia sede Bucaramanga y que cursaban la asignatura de Bases de Biogenética. El test instrumental fue leído y explicado por los profesores participantes y aplicado a cada uno de los estudiantes voluntarios de cada uno de los tres grupos en los que estaban divididos administrativamente, esto con el fin de llevar un seguimiento a futuro del desarrollo de esta competencia.

### Instrumento

En este ejercicio académico se utilizó el Cociente de Empatía EQ (Empathy Quotient) propuesto por Simon Baron-Cohen y Sally Wheelwright^15^. Este instrumento fue seleccionado sobre otros test de empatía como la Escala de Jefferson para estudiantes de enfermería (JSPE) debido a su fácil aplicabilidad y puntuación, además no separa ítems de categoría afectiva de categoría cognitiva, siendo esto muy importante ya que en el proceso empático los dos componentes coocurren de manera simultánea. Además, es importante reconocer nuestra visión de empatía desde el punto de vista biológico, como una herencia evolutiva que se construye con las variables ambientales que rodean al ser humano en su desarrollo, por lo que, al ser un test de amplio espectro, elimina el sesgo en el que puede influir la profesión. El instrumento consta de 60 items divididos en dos grupos: 40 ítems que evalúan empatía y 20 que operan como distractores.

El instrumento originalmente se calibró mediante el estudio de pacientes con el espectro autista ya que estos individuos presentan problemas en el desarrollo de habilidades sociales y comunicativas. El puntaje máximo obtenido a partir de los 40 items es de dos puntos por cada uno si la respuesta es “completamente de acuerdo” y de un punto si la respuesta al ítem es “ligeramente de acuerdo”. Valores por debajo de 20 puntos se correlacionan con el síndrome de Asperger y el autismo funcional, es decir, con muy pocas habilidades empáticas. El puntaje máximo a obtener es de 80 puntos. Es importante resaltar que con este instrumento se evalúa la empatía humana y no la laboral que en cierto sentido genera más honestidad al responder el test.

### Aspectos éticos

A los participantes se les explicó el ejercicio, y se les dio a conocer el consentimiento informado, el cual fue explicado y diligenciado con firma por aquellos estudiantes que quisieron participar. En tal sentido y para la propuesta futura de un proyecto de investigación los Profesores tienen bajo su custodia los test al igual que los consentimientos informados de forma física.

Si bien los resultados obtenidos surgen de un ejercicio académico como proyecto de aula, aportaron la información necesaria para la ejecución de procesos científicos de análisis. Es importante recalcar que estos resultados, su análisis y discusión no se sometieron al consejo de ética de la Facultad de Enfermería de la Universidad Cooperativa pues no se originan de un proyecto de investigación ni hacen parte de un proyecto mayor, sino que son el producto de actividades de enseñanza.

### Análisis de resultados

Con los resultados de los puntajes obtenidos se elaboró una escala de valores de empatía con base en el sexo y la edad. Se midieron las relaciones de las variables y su influencia en los niveles de empatía mediante pruebas de hipótesis como T y F y paramétricas ANOVA y no paramétricas Kruskal-Wallis para corroborar el análisis de distribución normal, dicha normalidad de los datos se midió con el test de Shapiro-Wilks con un Alfa de 0,05 (p > 0,05 acepto Ho: normalidad) utilizando el software estadístico de libre distribución PAST (https://www.nhm.uio.no/english/research/infrastructure/past/).

## Resultados

**Figura 3:**
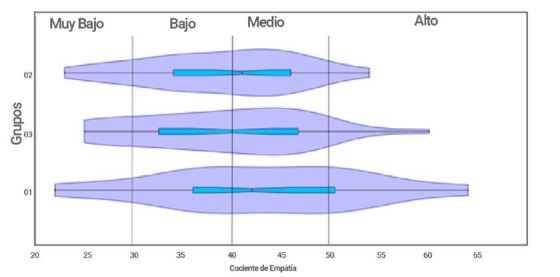
Cocientes de empatía en cada uno de los tres grupos del curso de Bases de Biogenética

Cien estudiantes del primer semestre de enfermería participaron en el test de empatía, con un rango de edades de 16 a 40 años, 25 hombres (25%), 74 mujeres (74%) y un ND (1%), ubicados entre los estratos sociales 1 al 4. El test de normalidad de Shapiro-Wilks mostró que los datos se ajustan a dicha distribución: Grupo 1 W: 0,97 y p: 0,72; Grupo 2 W: 0,95 y p: 0,18; Grupo 3 W: 0,97 y p: 0,52. Para la población total fue de W: 0,98 y p: 0,19. La figura 3, permite observar la distribución de los datos de empatía mediante curvas de densidad (KDE Kernel Density Estimate) y el diagrama de bigotes que indica la mediana, el valor máximo y el mínimo. Es posible determinar diferencias en los cocientes de empatía al interior de los grupos establecidos administrativamente, la media para el grupo 1 fue de 43 y para los otros dos cursos de 39, de igual forma se obtuvo un valor mínimo de 22 y un máximo de 64 en el cociente de empatía para esta población (total de los tres grupos). La mayor concentración de estudiantes según el cociente de empatía se encuentra para cada grupo en los rangos de: 30 a 48 para el grupo 2, de 25 a 48 para el grupo 3 y de 30 a 52 para el grupo 1 aproximadamente (Figura 3). Los valores mínimos y los máximos (extremos) constituyen una baja representatividad en la población de estudiantes de primer semestre.

**Figura 4:**
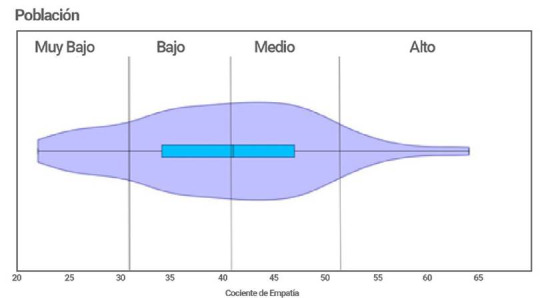
Clasificación poblacional según los cocientes de empatía

La Figura 4 muestra la distribución poblacional, es decir de todos los estudiantes con base en el cociente de empatía y la clasificación según los valores obtenidos. La mayor parte de los estudiantes se encuentran en niveles bajos y medios de empatía, con una cola en la gráfica bastante considerable de valores muy bajos.

**Figura 5:**
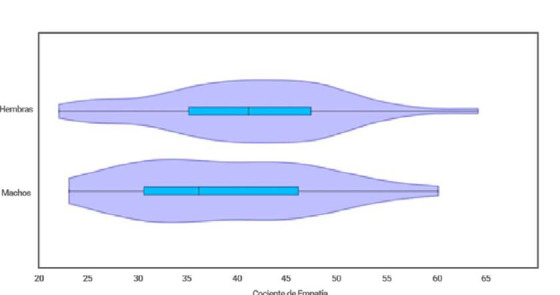
Cocientes de empatia en hombres y mujeres. Diferencias entre hombres y mujeres

El Análisis de Varianza para determinar homogeneidad de la empatía entre grupos se realizó para identificar si uno de los grupos de estudiantes presentaba valores significativos en la empatía y poder determinar el por qué. En este sentido no se hallaron diferencias significativas en el ANOVA entre los tres grupos obteniéndose una F de 2,098 (F0,05,(1), 2,97 = 3,10) y una p de 0,12. De igual forma la prueba no paramétrica de Kruskal-Wallis muestra que no hay diferencias entre los tres grupos con un Hc estadístico de 3,688 y una p de 0,15 (X2 0,05, 2 = 5,991).

La población de hombres en el primer semestre corresponde al 25% de la población, sin embargo, se realizaron dos test para evaluar las diferencias entre los sexos: la prueba de t (media) y la prueba F (varianza). Los resultados muestran que no hay diferencias significativas en los cocientes de empatía entre sexos, con una t de 1,0115 (t crítico de 1,98) y una F de 1,096 (F crítico de 1,83) ambos con un alfa de 0,05. La distribución de los valores de empatía en los dos sexos se aprecia en la Figura 5. La mayor parte de las mujeres se encuentran en los niveles de empatía bajos a medios, mientras que en los hombres se agrupan en los rangos de muy bajo a medio. Hombres y mujeres tienen pocos valores en el nivel alto. De hecho, las medias de cada sexo fueron 41 para mujeres y 38 para hombres.

### Relación de la empatía con la edad

**Figura 6:**
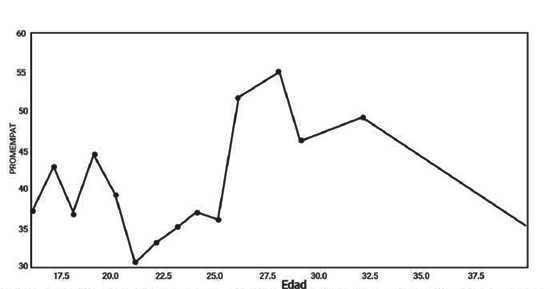
Variación del cociente de empatía a través de la edad

**Figura 7:**
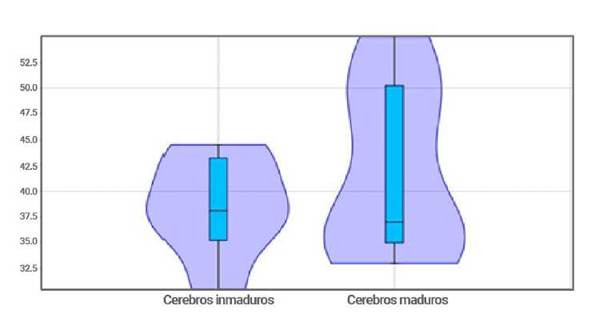
Empatía y madurez cerebral

Los resultados muestran que no existen diferencias estadísticas entre los grupos de estudiantes menores de 21 años y los mayores de 21 años (t de 0,90 y p de 0,38 para las medias estadísticas y de F de 2,83 y p de 0,17 para las varianzas) en los cocientes de empatía, si bien es cierto, pudiese apreciarse visualmente una tendencia que va en incremento, la curva cae al final debido a los valores bajos de únicos estudiantes mayores de 30 años, generando un sesgo estadístico (Figura 6). No se pudo explicar la relación entre el cociente de empatía y la edad con el modelo lineal, potencial o cuadrático. La Figura 7, muestra la distribución de los cocientes de empatía en las dos poblaciones artificiales creadas a partir de la edad y el concepto de que el cerebro madura completamente alrededor de los 21 a 25 años. Aunque estadísticamente no ha y diferencias, los patrones de distribución son diferentes.

Se aprecia la concentración de los estudiantes jóvenes menores de 21 años en el rango de 35 a 45 de empatía. La distribución de las personas con cerebros maduros es más elongada mostrando una tendencia a valores más altos de empatía.

### Relación de la empatía con el estrato social

**Figura 8:**
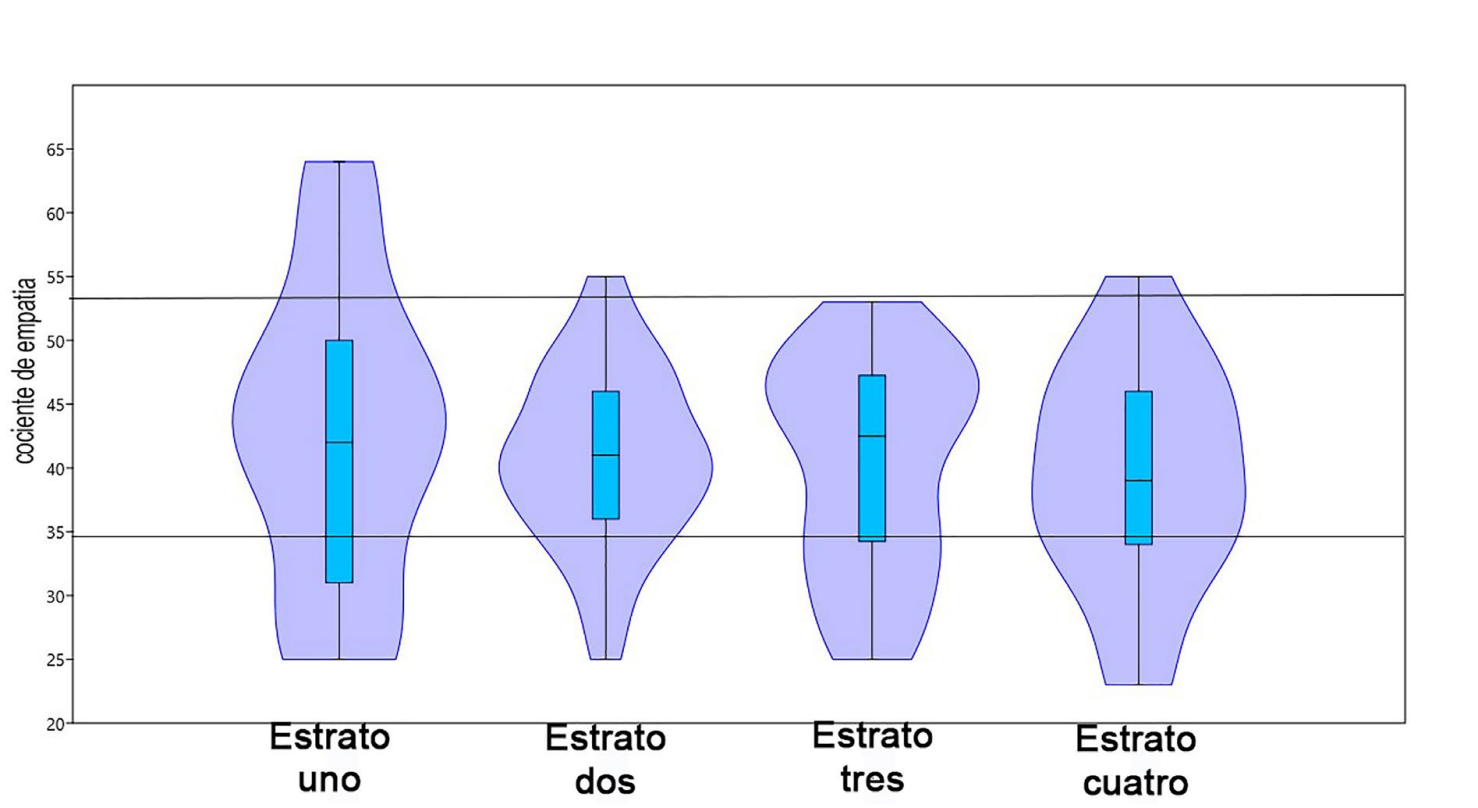
Distribución de los cocientes de empatía en cada uno de los estratos sociales Empatía y procedencia

No se hallaron diferencias significativas en el ANOVA entre los cuatro estratos sociales obteniéndose una F de 0,33 (F0,05,(1), 3,83 = 2,72) y una p de 0,79. De igual forma la prueba no paramétrica de Kruskal-Wallis muestra que no hay diferencias entre los cuatro grupos con un Hc estadístico de 0,68 y una p de 0,87 (X2 0,05, 3 = 7,815). La Figura 8, permite ver la distribución de los cocientes de empatía en los cuatro estratos sociales, observándose que el estrato uno cubre todos niveles de empatía desde los muy bajos hasta los altos, con una concentración en el rango de 35 a 54, el estrato dos presenta su máxima concentración entre los 33 y 50, el estrato tres una franja entre los 40 y los 52, y el estrato cuatro entre los 30 y los 50.

**Figura 9:**
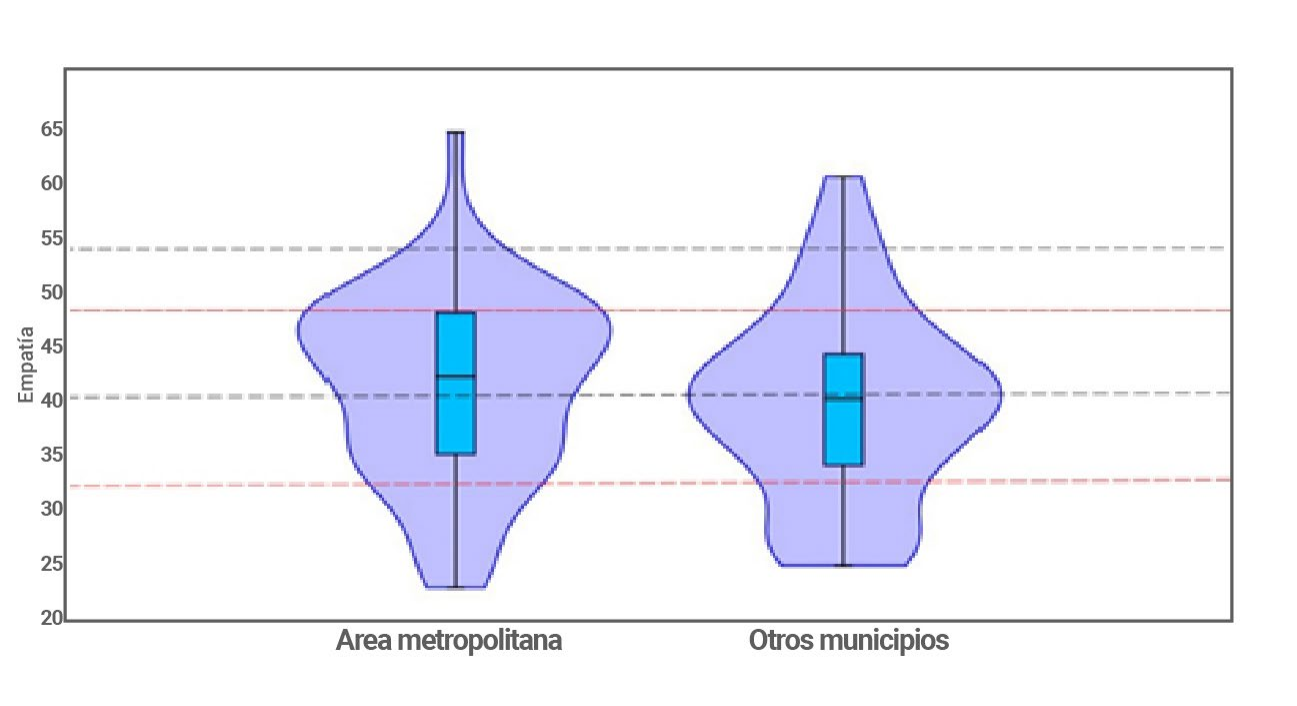
Distribución de los valores de empatía en el área metropolitana y otros municipios de procedencia de los estudiantes de primer semestre de Enfermería

Se analizaron los datos de procedencia probando la igualdad de medias o varianza entre el grupo de los municipios del área metropolitana (Bucaramanga, Girón, Floridablanca y Piedecuesta) y el grupo de municipios fuera del área metropolitana. No se encontraron diferencias entre los cocientes de empatía según la procedencia, con una t de 0,87 y una p de 0,38 (T crítico de 1,98) y una F de 1,08 y p de 0,77 (F critico de 1,83). La Figura 9, permite observar que la distribución de los valores de empatía en los otros municipios está en un rango menor al del área metropolitana.

## Discusión y Conclusiones

Frans de Waal en su libro La edad de la Empatía^16^ sostiene que “la empatía se despierta con más facilidad hacia los conocidos.. .de vez en cuando, también puede aplicarse fuera de este círculo interior.. .pero en general, la psicología primate está diseñada para preocuparse por el bienestar de la familia, los amigos y los compañeros. Por esta razón, los humanos también tienen respuestas contraempatéticas, por ejemplo, al saludar y sonreír a un estudiante, es típica la sonrisa falsa de gesto torcido, en palabras ajustadas de de Waal “como si nuestro placer les molestara”. Iniciamos la discusión con este párrafo para ejemplificar por qué utilizamos un estimador de empatía como el EQ, para evitar el sesgo de la presión social de la profesión “como soy enfermero o enfermera soy muy empático”, pues al evaluar la empatía en estudiantes de enfermería, la presión social del oficio, dirigirá las respuestas para tratar de sobrevalorar los niveles de la misma.

La media del cociente de empatía de la población de estudiantes de enfermería de primer semestre fue de 40,3 ubicándola en una posición intermedia, pero con una varianza muy grande de 82,4 que hace al grupo bastante heterogéneo, ubicándose gran parte de esta comunidad entre 30 y 50, es decir con un desarrollo de empatía bajo a medio.

No se encontraron diferencias significativas en las variables sexo, edad, estrato social y procedencia, se aprecian distribuciones que indican variables tensoras que pueden estar afectando el desarrollo de esta dimensión o competencia. Resultados similares obtuvieron Iratxe y Herrero-Fernández^17^^)^ quienes utilizando el EQ no confirmaron una mayor capacidad empática en la muestra de mujeres en su estudio con 471 estudiantes universitarios del País Vasco (74.4% mujeres) y de igual forma concluyeron que el EQ es una medida fiable y válida para evaluar empatía en muestras no clínicas.

Arciniegas-Arias^18^ en su tesis de grado de Psicología de la Universidad Cooperativa de Colombia publicada en el año 2020 y dirigida por Dora Cristina Cañas Betancur, establecen los niveles de empatía de los estudiantes de enfermería de la Universidad Cooperativa de Colombia sede Bucaramanga utilizando el TECA, Test de Empatía Cognitiva- Afectiva, con una muestra muy reducida, mal distribuida y sin respetar sus propios criterios de inclusión y exclusión, de estudiantes de los ocho semestres (n= 115) de este programa académico, concluyendo por ejemplo, con un gran sesgo que los estudiantes de primer nivel (n= 5) presentan niveles medios y que los de ultimo nivel (n= 15) valores altos. De igual forma concluyen erróneamente que hay diferencias significativas entre los niveles de empatía entre hombres y mujeres “podemos observar que la diferencia entre ambos sexos es mínima, para el sexo masculino es de 3,2 y para el sexo femenino es de 3,3 cabe resaltar que a pesar de parecer una diferencia pequeña en realidad una décima es muy importante”. Estos valores tendrían un valor de Chi2 de cero por lo que no existen diferencias en los resultados de estos autores.

En cuanto a la relación del nivel de empatía con el estrato social, Arciniegas-Arias^18^ concluye que la empatía se hace gradual a medida que se incrementa el nivel socioeconómico, en contraposición a los hallazgos obtenidos en este trabajo con estudiantes de primer semestre del mismo año donde no se encontraron diferencias significativas entre los estratos, los valores más altos de empatía se encontraron el estrato uno (Rango de 35-54) y el estrato cuatro registro los valores más bajos de empatía (Rango de 30-50).

Los análisis permitieron inferir que los niveles o valores en los cocientes de empatía dependen de otras variables distintas al sexo, la edad, estrato social o lugar de procedencia. La empatía según se observó, es una competencia que viene en un nivel básico en los adolescentes, con algunas excepciones, sigue su proceso de construcción hasta la completa formación de las redes neuronales o madurez cerebral alrededor de los 25 años^19^, donde estos neurocircuitos y la mielinogénesis siguen en construcción en el SNC regulados por las hormonas sexuales.

Se debe considerar también que el estrés físico, mental, económico y psicosocial, el abuso de drogas como el alcohol y las hormonas como los estrógenos, la progesterona y testosterona, afectan el desarrollo cerebral, por lo que se recomienda considerar el uso y abuso de medidas hormonales anticonceptivas en adolescentes en el desarrollo de la empatía.

La mayoría de estudiantes de primer semestre analizados están en sus procesos de madurez cerebral y lo más importante en la fase más aguda de plasticidad cerebral, donde el adolescente aprende y toma decisiones, lo que disminuye su capacidad de pensamiento crítico y racional^20^.

Según lo observado en este trabajo, es en este punto de “madurez cerebral” donde se aprecian caídas y aumentos en los cocientes de empatía que de manera inferencial deben estar dirigidos por factores ambientales experienciales. Si es la experiencia la que garantiza el correcto desarrollo de esta competencia debe ser crucial la intervención de las escuelas y facultades de enfermería con programas, experiencias aplicadas y ejercicios para producir profesionales competentes en esta dimensión. Razón de más, pues esos profesionales con edades entre 15 y 17 años obtendrán sus títulos cuando apenas ha finalizado la madurez cerebral o no la han alcanzado.

A diferencia de otros estudios con otros índices para evaluar empatía en estudiantes y personal del área de salud como el de González et al.^21^ o Díaz -Narváez et al.^11^, no se encontraron diferencias entre sexos, ni son las mujeres las que puntúan en esta escala. De igual forma los niveles moderados de empatía también fueron reportados por Ouzouni y Nakakis^10^ utilizando la Escala de Jefferson para estudiantes de enfermería (JSPE), de igual forma consideraron que la empatía se podía incrementar con educación experiencial apropiada.

Triana-Restrepo^19^ establece que los altos niveles de empatía que manifiestan las enfermeras se articulan con un menor estrés en los pacientes de cáncer, está de acuerdo con que la empatía es una competencia humana que puede modificarse y que puede ser enseñada efectivamente. Consideramos que este tipo de afirmaciones justifica el monitoreo continuo de esta dimensión o competencia desde los primeros niveles de formación universitaria.

Uno de los aportes significativos de este ejercicio académico es que busca acercarse a la realidad de los niveles de empatía de una persona desde una forma más neutra como individuo y no desde una postura profesional, en otras investigaciones los test de empatía aplicados a estudiantes o profesionales de la salud podrían generar un sesgo, debido a que el participante asume una postura profesional bajo la premisa de ser evaluado.
